# Les fistules coronaires: à propos d'un cas et revue de la literature

**DOI:** 10.11604/pamj.2015.21.145.7149

**Published:** 2015-06-23

**Authors:** Abdelmaji Bouzerda

**Affiliations:** 1 Service de Cardiologie 1, Centre Médicochirurgical, Agadir, Maroc

**Keywords:** Fistule coronaire, anomalie coronaire, chirurgie cardiaque, coronarographie, coronary fistula, coronary anomaly, heart surgery, coronary angiography

## Abstract

Les fistules coronaires sont des anomalies congénitales ou acquises relativement rares, au travers desquelles le flux sanguin coronaire est drainé vers une chambre cardiaque, un gros vaisseau ou une autre structure. Une cavité réceptrice à basse pression est souvent le site de drainage de ces fistules. Nous rapportons le cas d'un homme âgé de 30ans présentant des précordialgies atypique. L’épreuve d'effort est positive. L’évaluation par coronarographie met en évidence un réseau coronaire sain et un aspect de fistule coronarocamérale. L'objectif de cet article est d'aborder Les aspects cliniques, diagnostiques et thérapeutiques des fistules coronaires.

## Introduction

Pour la première fois décrites en post mortem par Krauss en 1865. Les fistules coronaires sont une communication anormale entre une artère coronaire et une cavité cardiaque ou un gros vaisseau de la base, court-circuitant ainsi le lit capillaire myocardique [1, 2]. Il s'agit d'une anomalie rare et généralement isolée [1, 3]. Aujourd'hui entre 0,1% et 0,5% des coronarographies a visée diagnostic révèlent la présence d une fistule coronaire. Tout cardiologue sera donc potentiellement confronte un jour à cette anomalie anatomique.

## Patient et observation

**Figure 1:**
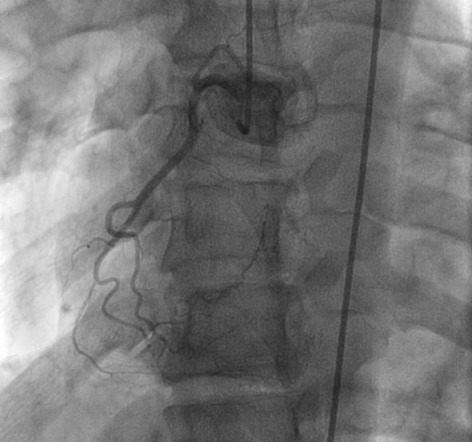
Incidence face craniale montrant un réseau coronaire droit dominé et sain

**Figure 2:**
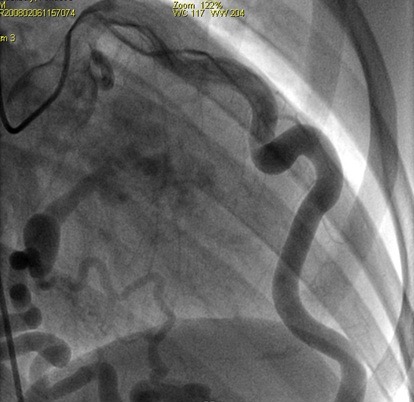
Incidence OAD craniale montrant un réseau interventriculaire antérieur éctasique, sain avec un aspect de fistule coronarocaméra

Mr.A.C. âgé de 30 ans consulte pour des précordialgies atypiques sans lien avec l'effort apparaissant depuis quelques mois. Ses facteurs de risque cardiovasculaire se résument à un tabagisme ancien chiffré à 10 PA. Son examen clinique d'admission note une Fréquence cardiaque à 75 battements par mn et une tension artérielle à 130/ 70 mm hg. L'auscultation cardiopulmonaire est normale, notamment pas de signes périphériques d'insuffisance cardiaque. Le reste de l'examen somatique est sans anomalie. L’électrocardiogramme de surface inscrit un rythme régulier sinusal à 75 cycles par minutes sans troubles de repolarisation ni hypertrophie pariétale. L'Echocardiographie transthoracique de repos montre un VG non dilaté normo cinétique avec une bonne fonction systolique VG (FE à 65%). Le doppler couleur note un flux longeant la paroi inférieure et antérolatérale et s'abouchant dans le VG, systolodiastolique. Le test d'effort sur cycloérgométre permet d'explorer plus de 85% de la réserve coronaire est positive cliniquement et électriquement à type de sous décalage ST de 2 mm en latéral. La coronarographie (Figure 1, Figure 2) réalisée par voie fémorale droite montre un réseau coronaire droit dominé et sain, un réseau gauche dominant avec une artère interventriculaire antérieure éctasique et un aspect de fistule coronarocamérale. La raction d’éjection ventriculaire gauche est normale sans troubles de la cinétique segmentaire à la ventriculographie. Nous avons soulevé l'indication d'une fermeture percutanée mais celle ci a été différer sur demande du patient qui préfère une surveillance annuelle.

## Discussion

Les fistules des artères coronaires (FC) sont des malformations rares faisant communiquer une artère coronaire avec une cavité cardiaque ou un gros vaisseau de la base [1]. L'incidence de ces anomalies est très faible et est retrouvée dans environ 0.02% de la population générale et de 0.5% de l'ensemble des patients ayant eu une coronarographie [3, 4]. La majorité des fistules sont congénitales, mais peuvent être acquises suite a une chirurgie cardiaque (notamment les remplacements valvulaires et les pontages aortocoronaires) ou à des biopsies myocardiques à répétition dans le cadre de transplantation cardiaque [5, 6]. Les fistules coronaires peuvent prendre naissance a partir des trois artères coronaires principales, y compris du tronc commun. L'artère coronaire droite est le plus souvent à l'origine de ces fistules (55%) contre 35% à partir de l'artère coronaire gauche, l'artère circonflexe étant plus rarement incriminée [7, 8]. Le mécanisme physiopathologique des FC sur cœur normal repose sur le vol du flux sanguin du territoire aval par la connexion fistuleuse pathologique. Cette réduction de perfusion est fonction du gradient de pression diastolique qui se crée entre le lit coronaire et la cavité réceptrice. Quelle que soit la coronaire concernée, la majorité de ces fistules se drainent dans la circulation veineuse, fonctionnant avec un régime de basses pressions: cavités droites du cœur, l'artère pulmonaire la veine cave supérieure et le sinus coronaire [9, 10]. Le drainage dans le VD est la modalité la plus fréquente soit 41% des cas [9]. L'importance du schunt est déterminée, d'une part, par la taille de la fistule et, d’ autre part, par la différence de pression entre la circulation systémique et la chambre cardiaque drainant celle-ci. La majorité des patients sont asymptomatiques. Ils sont généralement référés pour un souffle cardiaque de découverte fortuite.

Chez les patients les plus âgés, certains signes peuvent se voir: dyspnée d'effort, angor d'effort, arythmie, endocardite, accident vasculaire cérébral [11, 12]. En cas de shunt gauche droit large, des complications peuvent avoir lieu: hypertension artérielle pulmonaire, insuffisance cardiaque congestive, dilatation anévrysmale, infarctus myocardique par un phénomène de vol artériel [13]. Plusieurs modalités d'imagerie existent pour le diagnostic et surtout pour préciser l'anatomie exacte du trajet fistuleux. L’Écho doppler cardiaque, notamment transœsophagienne est un examen utile pour le diagnostic des fistules à large shunt gauche droite. En effet une artère coronaire dilatée ainsi que son site de drainage peuvent être détectée. Dans certains cas une perturbation du signal Doppler est mise en évidence au site de drainage surtout ventriculaire droit. Le scanner cardiaque apporte des informations d'ordre morphologique concernant la fistule: réseau coronaire touché, calibre de l'artère nourricière, diagnostic des complications. L'examen de référence est la coronarographie qui permet de confirmer le diagnostic et d’étudier l'anatomie exacte de la fistule coronaire en mettant en évidence l'artère concernée, qui est souvent dilatée, le trajet fistuleux et le site de drainage. Le pronostic est lié aux complications. La fermeture spontanée est rare et concerne 23% des fistules de petit calibre et surtout dépendante du réseau coronaire gauche.

En raison de l’évolution anévrysmale naturelle mais lente des FC, l'insuffisance cardiaque congestive, les infarctus myocardiques et les complications anévrismales sont plutôt tardifs. La stratégie de prise en charge repose sur la fermeture de ces fistules coronaires soit par voie chirurgicale soit par voie percutanée. Le traitement historique est la ligature chirurgicale, pour la première fois réalisée par Bjork et Crawford en 1946 en l'absence de circulation extracorporelle, elle peut être réalisée par voie épicardique, endocardique ou combiner les deux. La fermeture peut être directe ou réalisée à l'aide d un patch dans le cas d'une large communication entre la coronaire et la cavité cardiaque. Le traitement chirurgical montre une excellente efficacité et sécurité à long terme. Il sera le traitement de choix des fistules multiples ou volumineuses, ou des fistules associées à d'autres anomalies cardiaques. La fermeture percutanée est également un moyen fiable avec de bons résultats [14, 15]. Les matériaux utilisés sont variés. Dans plus de 70% des cas, coils métalliques, ballonnets largables et système de type ombrelle et plus rarement des stents couverts qui permettent l'exclusion de la fistule et dont l'indication est réduite aux situations d'urgence ou lorsque la coronaire est protégée par un pontage. Les fistules de plus gros diamètres requièrent l'utilisation d'autres systèmes d'occlusion comme des Plugs vasculaires oversizés ou des amplatzers.

## Conclusion

Les fistules coronaires représentent une anomalie de terminaison coronaire rare dont l’évolution est souvent défavorable à long terme. leur diagnostic morphologique est accessible: scanner cardiaque, coronarographie qui permettent une caractérisation précise et une planification préthérapeutique complète. L'approche thérapeutique va dépendre de la situation anatomique. Si l'abord chirurgical reste valide, l'occlusion percutanée semble actuellement le choix thérapeutique le plus approprié.

## References

[1] Shakeel AQ (2006). Coronary arterial fistulas. Orphanet J Rare Dis..

[2] Gowda RM, Vasavada BC, Khan IA (2006). Coronary artery fistulas: clinical and therapeutic considerations. Int J Cardiol..

[3] Shiga Y (2008). Left main coronary trunk connecting into right atrium with an aneurismal coronary artery fistula. Int J Cardiol..

[4] Vavuranakis M, Bush CA, Boudoulas H (1995). Coronary artery fistulas in adults: incidence, angiographic characteristics, natural history. Cathet Cardiovasc Diagn..

[5] Reidy JF, Anjos RT, Qureshi SA, Baker EJ, Tynan MJ (1991). Transcatheter embolization in the treatment of coronary artery fistulas. J Am Coll Cardiol..

[6] Somers JM, Verney GI (1991). Coronary cameral fistulae following heart transplantation. Clin Radiol..

[7] Lopez-Candales A, Kumar V (2005). Coronary artery to left ventricle fistula. Cardiovasc Ultrasound..

[8] Umana E, Massey CV, Painter JA (2002). Myocardial ischemia secondary to a large coronary-pulmonary fistula - a case report. Angiology..

[9] Levin DC, Fellows KE, Abrams HL (1978). Hemodynamically significant primary anomalies of the coronary arteries. Circulation..

[10] Fujimoto N, Onishi K, Tanabe M (2004). Two cases of giant aneurysm in coronary pulmonary artery fistula associated with atherosclerotic change. Int J Cardiol..

[11] Balanescu S, Sangiorgi G, Castelvecchio S, Medda M, Inglese L (2001). Coronary artery fistulas: clinical consequences and methods of closure: a literature review. Ital Heart J..

[12] Wang NK, Hsieh LY, Shen CT, Lin YM (2002). Coronary arteriovenous fistula in pediatric patients: a 17-year institutional experience. J Formos Med Assoc..

[13] Oshiro K, Shimabukuro M, Nakada Y, Chibana T, Yoshida H, Nagamine F (1990). Multiple coronary LV fistulas: demonstration of coronary steal phenomenon by stress thallium scintigraphy and exercise haeodynamics. Am Heart J..

[14] Reidy JF, Jones ODH, Tynan MJ, Baker EJ, Joseph MC (1985). Embolization procedures in congenital heart disease. Br Heart J..

[15] Perry SB, Rome J, Keane JF, Baims DS, Lock JF (1992). Transcatheter closure of coronary artery fistulas. J Am Coll Cardiol..

